# Temporal impact of metritis and systemic antibiotics on immune cell function inferred from the blood leukocyte transcriptome

**DOI:** 10.3168/jdsc.2026-1067

**Published:** 2026-06-17

**Authors:** Monica O. Caldeira, Joao G.N. Moraes, Scott E. Poock, Matthew C. Lucy

**Affiliations:** 1 Division of Animal Sciences, University of Missouri, Columbia, MO 65211; 2 Department of Animal and Food Sciences, Oklahoma State University, Stillwater, OK 74078; 3 College of Veterinary Medicine, University of Missouri, Columbia, MO 65211

## Abstract

Within 2 wk postpartum approximately 20% of dairy cows develop metritis, a uterine infection associated with reduced fertility and increased risk of reproductive culling. Antibiotics improve clinical cure and milk production following metritis but do not consistently increase subsequent fertility postpartum compared with untreated control cows. To better understand why antibiotic treatment improves clinical recovery but not fertility, we tracked changes over time in blood leukocyte gene expression to examine circulating immune cell function during the resolution of metritis in antibiotic-treated and untreated cows. Twenty-three primiparous Holstein cows diagnosed with metritis (n = 11) or classified as healthy controls (n = 12) between 7 and 10 d postpartum were enrolled. Cows were assigned to receive systemic ceftiofur (1.25 g/d for 3 d) or remain untreated in a 2 × 2 factorial design. Blood samples were collected at diagnosis (wk 1), and at 1 and 3 wk after treatment (wk 2 and wk 4) postpartum. The blood leukocyte RNA was isolated and sequenced, and differentially expressed genes were analyzed for functional enrichment and immune cell type. Healthy cows exhibited enrichment of B cell activation and receptor signaling pathways, whereas metritis cows showed upregulation of inflammatory and innate immune responses, including monocyte and neutrophil markers. In untreated cows (healthy versus metritis), transcriptomic differences in blood leukocytes were resolved by wk 4 postpartum. In contrast, antibiotic-treated metritis cows displayed a distinct immune trajectory. At wk 2 postpartum (1 wk after antibiotic treatment), cytotoxic T cell and natural killer cell gene expression signatures increased, differing clearly from untreated metritis cows. By wk 4, a second wave of inflammatory cell–associated differentially expressed genes emerged exclusively in antibiotic-treated metritis cows, suggesting prolonged immune cell activation. These findings demonstrate that antibiotic treatment reshapes systemic immune dynamics during recovery from metritis. The altered sequence (e.g., early cytotoxic activation followed by extended inflammation) may explain why antibiotic-induced clinical cure in metritis cows does not consistently translate into improved fertility.

## Highlights

• The postpartum blood leukocyte transcriptome differed for healthy and metritis cows.
• Antibiotics changed the blood leukocyte transcriptome of metritis cows within 1 week.
• Antibiotics prolonged the inflammatory response to metritis in blood.

## Graphical Abstract

**Figure:**
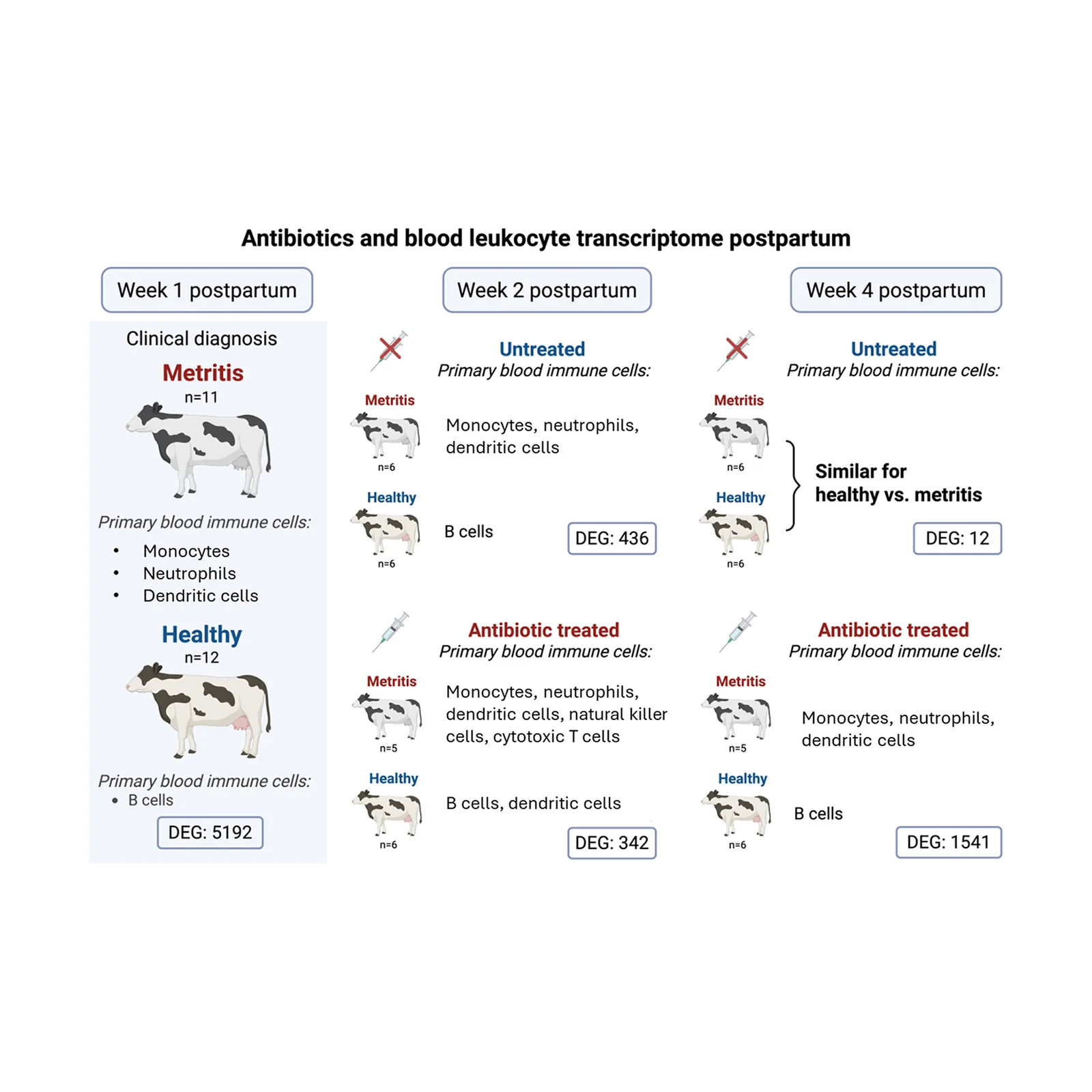
**Summary:** We tested immune resolution of metritis in antibiotic-treated and untreated cows by analyzing the blood leukocyte transcriptomes at 1 and 3 weeks after diagnosis and treatment. The blood leukocyte transcriptome of 23 first-lactation Holstein cows (healthy or with metritis; treated or untreated) was analyzed by using RNA sequencing and differential gene expression analyses. Healthy cows had blood leukocyte transcriptomes indicative of B lymphocytes. Cows diagnosed with metritis had blood leukocyte transcriptomes of inflammatory myeloid cells and cytotoxic lymphocytes. Untreated metritis cows gradually returned to a healthy-like transcriptome within 3 weeks. In contrast, antibiotic-treated metritis cows displayed a distinct trajectory (based on blood leukocyte transcriptome) that included an initial wave of cytotoxic T and natural killer cells (1 week after treatment) followed by a second wave of inflammatory cells (3 weeks after treatment). These findings indicate that antibiotics redirect, rather than replicate, the temporal sequence of immune cell populations involved in disease recovery. Created with BioRender.com.

Approximately 20% of dairy cows develop metritis (uterine infection) within 2 wk after calving (Wagener et al., 2017; Pérez-Báez et al., 2021; Garzon et al., 2022). The infection likely arises from bacteria of the gastrointestinal tract ascending into the uterus from the lower genital tract (Jones et al., 2022). Metritis places the cow at risk for subfertility, which increases the likelihood of reproductive culling (Moraes et al., 2021; LeBlanc, 2023; Bruinjé et al., 2024). Metritis cows are commonly treated with antibiotics because antibiotics increase the likelihood of clinical cure (by reducing fever and vaginal discharge score) and improve milk production (de Oliveira et al., 2020; Menta et al., 2024). Although antibiotics improve clinical cure rates, evidence that they shorten the time to pregnancy postpartum is inconsistent (de Oliveira et al., 2020; Menta et al., 2024). In a recent study by Pereira et al. (2025), for example, cows that showed clinical cure (a reduction in vaginal discharge) were more likely to become pregnant. Antibiotic treatment increased the risk of clinical cure, but did not increase the likelihood of pregnancy (Pereira et al., 2025). We hypothesized that the resolution of inflammation associated with metritis differs between antibiotic-treated and untreated cows. To test this, we collected blood samples, isolated leukocytes, and analyzed gene expression (the blood leukocyte transcriptome) to estimate the function of immune cells in circulation at 1 and 3 wk after treatment (i.e., 2 and 4 wk postpartum). A different blood leukocyte transcriptome in treated and untreated cows would indicate that the immune response and disease recovery and trajectory depend on whether antibiotics are used. This difference could help explain why clinical cure and pregnancy outcomes are uncoupled in antibiotic-treated cows.

The present study was a component of 2 studies that were previously published that described the microbiome (Moraes et al., 2025) and the biology and morphology (Caldeira et al., 2025) of the bovine uterus following antibiotic treatment of metritis. Twenty-three first-lactation Holstein cows were enrolled. Metritis was identified by the herdsman based on clinical signs at 7 to 10 d postpartum (fetid red-brown watery vaginal discharge with a flaccid uterus). The metritis diagnosis was confirmed by a veterinarian from the University of Missouri, who examined the uterus and uterine discharge of the selected cows. Cows diagnosed with metritis (n = 11) were matched with healthy control (n = 12) cows that calved within the same week. Cows were either antibiotic-treated (metritis-treated, n = 5, and healthy-treated, n = 6) with ceftiofur hydrochloride (i.m. 1.25 g/d for 3 d; Excenel RTU, Zoetis, Parsippany-Troy Hills, NJ) or left untreated (metritis-untreated, n = 6, and healthy-untreated, n = 6). Blood samples were collected weekly beginning at disease diagnosis and before treatment (wk 1; 7.3 ± 1.5 d postpartum), 1 wk after treatment (wk 2; 13.8 ± 1.4 d postpartum) and 3 wk after treatment (wk 4; 26.8 ± 1.6 d postpartum). Samples were collected from the coccygeal veins into a K_2_ EDTA tube (10 mL Monoject EDTA, Covidien, Mansfield, MA). After plasma separation (1,200 × *g* for 20 min at 4°C), the buffy coat was transferred into 12 mL of red blood cells lysis buffer (100 m*M* NH_4_Cl, 10 m*M* NaHCO_3_, 1 m*M* EDTA, pH 7.0), incubated for 5 min at room temperature and then centrifuged at 300 × *g* for 10 min at 4°C. The supernatant was discarded and the process repeated with 5 mL of lysis buffer. The pellet was washed with 5 mL of ice-cold 1× Dulbecco's PBS (ThermoFisher, Waltham, MA), and centrifuged. The leukocyte pellet was resuspended with 600 μL of RLT buffer (Qiagen, Germantown, MA) with 10% of 2-mercaptoethanol and kept at −80°C until RNA isolation. A modified TRIzol-chloroform protocol was used for RNA isolation as described in Study 2 of Silva et al. (2024). The RNA concentrations were measured using high-sensitivity RNA analysis. The RNA quality number (1 = degraded to 10 = fully intact) averaged 8.98 (SD = 0.79).

The MU Genomics Technology Core (Columbia, MO) performed RNA library preparation and sequencing. Stranded RNA-seq libraries were constructed using the poly-A enrichment method with the Illumina Stranded mRNA Prep (Illumina, San Diego, CA). Each purified library was sequenced on a NovaSeq 6000 sequencer (Illumina) using a single NovaSeq S2-PE100 flow cell with an average read count of approximately 60 million paired-end reads per sample (range: 46–78 million). Raw paired-end reads (2 × 100 bp) were trimmed using cutadapt (v3.2; Martin, 2011). Illumina TruSeq adapter sequences were removed from the 3′ ends of R1 (AGATCGGAAGAGCACACGTCTGAACTCCAGTCA) and R2 (AGATCGGAAGAGCGTCGTGTAGGGAAAGAGTGT) reads. Poly-G tails arising from empty flow cell tiles on the NovaSeq 6000 platform were trimmed from both reads, and ambiguous N bases were removed from read termini. Reads shorter than 10 bp following trimming were discarded. Ribosomal RNA content was low across all samples (average 2.4%; range: 1.0%–4.8%), confirming effective poly-A enrichment. A genome index was built from the Ensembl *Bos taurus* ARS-UCD1.2 reference genome using HISAT2-build (v2.1.0) with default parameters. Trimmed paired-end reads were then aligned to this index using HISAT2 (v2.1.0; Kim et al., 2015) with default parameters, achieving an average alignment rate of 94.1% per sample (range: 92.0%–95.5%). Subread's featureCounts (v2.0.0; Liao et al., 2014) was used to quantify reads mapped to exon sequences of annotated genes using the Ensembl *Bos taurus* ARS-UCD1.2 gene annotation file (release 102), with paired-end mode enabled (-p) and default parameters for all remaining settings. An average of 66.4% of aligned reads per sample were successfully assigned to annotated genes (range: 62.0%–70.8%), corresponding to an average of 42.2 million read pairs per sample. The resulting count matrix was then filtered using the filterByExpr function of edgeR (v3.36.0; Chen et al., 2016) with default parameters and normalized using the trimmed mean of M-values (TMM) method. Differentially expressed genes (**DEG**) were identified using edgeR by fitting a negative binomial generalized linear model with quasi-likelihood *F*-tests and robust dispersion estimation. A transcript was considered differentially expressed if the false discovery rate (**FDR**)-adjusted *P*-value was <0.05. The computer code for the RNA sequencing is available in the supplemental material (see Notes).

Blood leukocyte transcriptome analyses were based on the original assignment to the disease-treatment groups (wk 1 postpartum). Five group comparisons were performed within week: (1) metritis versus healthy, (2) untreated metritis cows versus untreated healthy cows, (3) antibiotic-treated metritis cows versus antibiotic-treated healthy cows, (4) antibiotic-treated healthy cows versus untreated healthy cows, and (5) antibiotic-treated metritis cows versus untreated metritis cows. Additional group comparisons based on disease status at 2 or 4 wk postpartum were not performed because of the small number of cows in some groups (healthy cows that developed endometritis, for example; Caldeira et al., 2025; Moraes et al., 2025). A transcript was defined as differentially expressed if the FDR-adjusted *P*-value was <0.05. The DEG lists were uploaded to the Database for Annotation, Visualization, and Integrated Discovery (DAVID v2024q4; https://davidbioinformatics.nih.gov/home.jsp/) to detect enriched Gene Ontology (**GO**) biological process (**BP**) direct terms (GO BP terms) and Kyoto Encyclopedia of Genes and Genomes (**KEGG**) pathways. Enrichment of GO terms and KEGG pathways were retained if the FDR-adjusted *P*-value was <0.05. Lists with DEG were then uploaded to ShinyGO 0.85 (http://bioinformatics.sdstate.edu/go/; Ge et al., 2020) to detect enriched cell types using the CellMarker.2024 database (http://www.bio-bigdata.center/; Hu et al., 2023) and the Enrichr program (https://maayanlab.cloud/Enrichr; Chen et al., 2013; Kuleshov et al., 2016; Xie et al., 2021).

In the analysis of all cows (metritis versus healthy), the greatest number of DEG was observed in wk 1 (5,192 DEG; Table 1; Supplemental Table S1A, see Notes). This number declined in wk 2 (1,424 DEG; Supplemental Table S1B) and wk 4 (1,286 DEG; Supplemental Table S1C). The DEG lists were then analyzed using DAVID to identify enriched GO BP terms and KEGG pathways. In wk 1 (Supplemental Table S2A, see Notes), genes that were downregulated in metritis cows (i.e., upregulated in healthy cows) were identified by the GO BP term “B cell activation” as well as numerous KEGG pathway immune function terms including “B cell receptor signaling.” In contrast, genes upregulated in metritis cows were identified by the GO BP terms “inflammatory response,” “innate immune response,” “regulation of inflammatory response,” “defense response to bacterium,” and “phagocytosis” in addition to a variety of immune system functions. Although the number of DEG decreased during wk 2 (Table 1), the enriched GO BP terms and KEGG pathways followed a similar pattern (B cell activation and signaling in healthy cows and inflammatory response in metritis cows; Supplemental Table S2B). The upregulated DEG in wk 4 in metritis cows (Table 1) were primarily associated with inflammation (Supplemental Table S2C).

**Table 1:** Number of DEG in RNA and predicted cell types in blood leukocytes collected from cows that were diagnosed as either healthy or having metritis at 7 to 10 d postpartum and either antibiotic-treated or untreated (control)

Item	Week 1	Week 2	Week 4
DEG for all cows by week^1^			
DEG increased in healthy cows versus metritis cows	2,267	432	734
DEG increased in metritis cows versus healthy cows	2,925	992	552
DEG for untreated cows (healthy, n = 6, and metritis, n = 6)			
DEG increased in untreated healthy cows versus untreated metritis cows		78	6
DEG Increased in untreated metritis cows versus untreated healthy cows		358	6
DEG for antibiotic cows (healthy, n = 6, and metritis, n = 5)			
Increased DEG in antibiotic-treated healthy cows versus antibiotic-treated metritis cows		87	788
Increased DEG in antibiotic-treated metritis cows versus antibiotic-treated healthy cows		255	753
DEG in healthy cows (antibiotic, n = 6, and untreated, n = 6)			
Increased DEG in untreated healthy cows versus antibiotic-treated healthy cows		7	3
Increased DEG in antibiotic-treated healthy cows versus untreated healthy cows		5	5
DEG in metritis cows (antibiotic, n = 5, and untreated, n = 6)			
Increased DEG in untreated metritis cows versus antibiotic-treated metritis cows		34	10
Increased DEG in antibiotic-treated metritis cows versus untreated metritis cows		28	13

1 Differentially expressed genes (DEG; FDR < 0.05) and fold change are listed in Supplemental Table S1A–K.

The blood leukocyte transcriptome mainly reflects RNA from immunocytes, which develop from hematopoietic stem cells that differentiate into the myeloid lineage (neutrophils, basophils, eosinophils, monocytes, and dendritic cells) and lymphoid lineage (T cells, B cells, and natural killer [**NK**] cells). Differentially expressed genes can be “deconvoluted” to estimate the relative function of these immune cell types using bioinformatic approaches (White et al., 2024). We used the CellMarker.2024 database and the Enrichr program to compare our DEG lists (increased or decreased DEG within our 5 group comparisons) with the gene-set libraries in the CellMarker.2024 database. The enrichment FDR was calculated to assess the significance of overlap between the input list and the gene sets in each gene-set library (Supplemental Table S3, see Notes). The overlapping gene lists (our DEG compared with the gene sets in each CellMarker.2024 gene-set library) included both definitive marker genes for specific immune cell types (Supplemental Table S4, see Notes) as well as shared genes (Supplemental Table S3).

Using the approach outlined in the preceding paragraph, we were able to infer changes in immune cell function defined by the DEG (Table 1). The function of circulating immune cells found in healthy cows (based on DEG) were related to B cells during wk 1 (Supplemental Table S3A) and wk 2 (Supplemental Table S3B). During wk 1 and 2, for example, the DEG list for healthy cows overlapped with 9 and 21 B cell gene-set libraries in CellMarker.2024. The DEG list in metritis cows overlapped with a single B cell gene-set during wk 1 (Supplemental Table S4). Selected B cell marker genes (*CD19*, *CD21*, *CD79A*, *CD79B*, *MS4A1*, and *PAX5*) had greater expression (log2 fold change) in the healthy cows during wk 1 compared with metritis cows (Table 2). The healthy cows also had greater expression for some of the cytotoxic and T cell marker genes (*GNLY*, *KLRK1*, *GZMM*, and *PRF1*; Table 2).

**Table 2:** Log_2_ fold change and FDR^1^ for genes whose expression is characteristic of individual immune cell types within DEG lists in Table 1 and Supplemental Table S1

Marker gene	Week 1 all cows (H vs. M)		Week 2 untreated cows (H vs. M)		Week 2 antibiotic cows (H vs. M)		Week 4 antibiotic cows (H vs. M)	
	Log_2_ fold change	FDR	Log_2_ fold change	FDR	Log_2_ fold change	FDR	Log_2_ fold change	FDR
B cell								
*CD19*	−0.888 (H)	6.54E-05	−0.549 (H)	4.18E-03	−0.626 (NS)	0.151	−0.908 (H)	8.51E-03
*CD21*	−0.893 (H)	4.61E-04	−0.745 (H)	8.19E-03	−0.716 (NS)	0.118	−0.631 (NS)	0.125
*CD79A*	−0.958 (H)	2.71E-05	−0.661 (H)	1.01E-03	−0.664 (NS)	0.158	−0.476 (NS)	0.260
*CD79B*	−1.011 (H)	4.52E-05	−0.675 (H)	5.04E-03	−0.766 (NS)	0.172	−0.765 (NS)	0.078
*MS4A1*	−1.037 (H)	2.07E-05	−0.673 (H)	1.00E-03	−0.823 (NS)	0.073	−0.814 (H)	0.039
*PAX5*	−0.871 (H)	4.64E-03	−0.457 (NS)	0.216	−0.680 (NS)	0.391	−1.000 (H)	1.96E-03
Monocyte								
*CD14*	1.349 (M)	2.81E-06	0.642 (M)	6.78E-03	0.584 (NS)	0.255	0.876 (M)	0.050
*CD68*	1.295 (M)	2.19E-05	0.737 (M)	6.31E-04	0.823 (M)	0.027	1.310 (M)	2.16E-03
*CD172*	1.146 (M)	4.85E-06	0.647 (M)	0.0162	0.626 (NS)	0.137	0.960 (M)	0.028
*CSF1R*	0.711 (M)	5.11E-03	0.617 (M)	9.96E-04	0.582 (NS)	0.180	0.817 (M)	0.018
*ITGAM*	0.590 (M)	1.32E-03	0.207 (NS)	0.453	−0.257 (NS)	0.605	0.297 (NS)	0.301
Cytotoxic T cells, NK cells								
*CTSW*	−0.416 (NS)	0.087	0.560 (M)	0.047	0.801 (M)	9.26E-03	0.308 (NS)	0.335
*GNLY*	−0.823 (H)	2.79E-03	0.500 (NS)	0.075	1.082 (M)	2.33E-03	0.606 (NS)	0.108
*KLRK1*	−0.655 (H)	1.22E-02	0.693 (M)	0.023	0.807 (NS)	0.115	−0.447 (NS)	0.396
*GZMM*	−0.759 (H)	1.40E-02	0.572 (NS)	0.152	0.988 (M)	4.19E-02	0.030 (NS)	0.962
*NKG7*	−0.307 (NS)	0.177	0.637 (M)	6.22E-03	0.675 (M)	4.07E-02	0.449 (NS)	0.139
*PRF1*	−0.684 (H)	5.91E-03	0.507 (NS)	0.063	0.693 (M)	3.47E-02	0.074 (NS)	0.855
Neutrophil								
*CD177*	6.847 (M)	1.05E-39	2.433 (M)	4.62E-04	1.918 (NS)	0.188	3.364 (NS)	0.061
*CSF3R*	1.286 (M)	5.54E-05	0.631 (M)	0.044	0.722 (NS)	0.175	0.714 (NS)	0.171
*IL2RA*	1.574 (M)	1.72E-08	0.394 (M)	0.047	0.431 (NS)	0.456	−0.091 (NS)	0.887
*MPO*	1.851 (M)	2.33E-04	−0.169 (NS)	0.868	0.726 (NS)	0.665	1.055 (NS)	0.428

1 For the healthy (H) versus metritis (M) comparison, a positive log_2_ fold change indicates greater expression in M versus H cows, whereas a negative log_2_ fold change indicates greater expression in H versus M cows. The direction of change (toward H or toward M; FDR < 0.05) is indicated within the parentheses. NS = not significant.

Based on overlap with the gene-set libraries in CellMarker.2024, the leukocytes present in the metritis cows for wk 1 (Supplemental Table S3A), wk 2 (Supplemental Table S3B) and wk 4 (Supplemental Table S3C) included diverse immune cell types including progenitor cells, cells from the myeloid lineage (monocytes, neutrophils, and dendritic cells) and cells from the lymphoid lineage (B and T cells, both CD4^+^ and CD8^+^, and NK cells). There were 33 versus 0 overlapping gene-set libraries for monocytes in metritis versus healthy cows respectively during wk 1 (Supplemental Table S4). There were 7 versus 0 overlapping gene-set libraries for neutrophils in metritis versus healthy cows (Supplemental Table S4). Monocyte marker genes (*CD14*, *CD68*, *CSF1R*, and *ITGAM*) and neutrophil marker genes (*CD177*, *CSF3R*, *IL2RA*, and *MPO*) were greater in metritis cows during wk 1 (Table 2).

The next analyses examined untreated and antibiotic-treated cows separately at wk 2 postpartum (1 wk after treatment) and wk 4 postpartum (3 wk after treatment). In untreated cows (healthy versus metritis) there were 436 DEG at wk 2 postpartum (Table 1; Supplemental Table S1D). The enriched GO BP terms and KEGG pathways indicated B cell signaling in healthy cows and phagocytosis, inflammatory response, and innate immune response in metritis cows; Supplemental Table S2D). The predominate cell types identified for the wk 2 untreated healthy cows (based on overlap with gene-set libraries in CellMarker.2024) were progenitor cells and B cells whereas the blood leukocyte of untreated metritis cows possessed progenitor cells, monocytes, dendritic cells, and neutrophils (Supplemental Table S3D). Analysis of individual log2 fold change of immune cell marker genes (Table 2) confirmed the presence of B cells in untreated healthy cows at wk 2 and monocytes and neutrophils in untreated metritis cows at wk 2 (Table 2). Two week later (wk 4), only 12 DEG were identified between untreated healthy cows and untreated metritis cows (Table 1; Supplemental Table S1E). This small number of DEG suggests that the circulating leukocyte profiles of untreated metritis cows were like those of healthy cows by 4 wk postpartum.

We next examined the blood leukocyte transcriptome of healthy and metritis cows treated with antibiotics (Table 1). The results of these analyses were distinctly different from those derived from the untreated cows. There were 342 DEG for the analysis of healthy antibiotic-treated cows versus metritis antibiotic-treated cows at wk 2 (1 wk after antibiotic treatment; Table 1; Supplemental Table S1F). There were 4 enriched GO BP terms and KEGG pathways in the antibiotic-treated metritis cows, 1 of which was defined as “killing of cells of another organism” (Supplemental Table S2E). Based on the overlap with gene-set libraries in CellMarker.2024, there were 2 cell types identified in the antibiotic-treated healthy cows at wk 2 (B cells and dendritic cells; Supplemental Table S3E). The wk 2 antibiotic-treated metritis cows possessed progenitor cells, monocytes, dendritic cells, neutrophils, B cells, T cells (cytotoxic, CD4^+^, and CD8^+^) and NK cells (Supplemental Table S3E). With respect to marker genes (Table 2), the wk 2 antibiotic-treated metritis cows were enriched for cytotoxic T cells and NK cells. Two weeks later (wk 4 postpartum), there were 1,541 DEG for antibiotic-treated healthy versus metritis cows (Table 1; Supplemental Table S1G). The enriched GO BP terms were “innate immune response” and “inflammatory response” in metritis cows (Supplemental Table S2F). There were no enriched terms for antibiotic-treated healthy cows at wk 4. The predominate cell types identified for the wk 4 antibiotic-treated healthy cows were B cells whereas the wk 4 antibiotic-treated metritis cows possessed progenitor cells, monocytes, dendritic cells, and neutrophils (Supplemental Table S3F). Marker genes for B cells (*CD19*, *MS4A1*, and *PAX5*) were increased in antibiotic-treated healthy cows at wk 4 and marker genes for monocytes (*CD14*, *CD68*, *CD172*, and *CSF1R*) were increased in antibiotic-treated metritis cows at wk 4 (Table 2).

In a final analysis, we compared the effects of antibiotic treatment within disease classifications (healthy or metritis). Within healthy cows, treatment with antibiotics had a minimal effect on the blood leukocyte transcriptome at either wk 2 (12 DEG total) or wk 4 (8 DEG total; Table 1). Likewise for metritis cows alone, there was a minimal effect of antibiotic treatment on the blood leukocyte transcriptome (62 DEG at wk 2 [1 wk after treatment] and 23 DEG at wk 4 [3 wk after treatment]; Table 1).

The RNA sequencing demonstrated clear differences in the blood leukocyte transcriptome for healthy versus metritis cows during the postpartum period. We found that compared with metritis cows, healthy cows had leukocyte gene expression consistent with B cells in their circulation. Metritis cows had leukocyte gene expression consistent with inflammatory cells in circulation (monocytes, neutrophils, NK cells, and cytotoxic T cells). The number of DEG for healthy versus metritis cows decreased over time (Table 1). In untreated cows (healthy versus metritis), there were more than 400 DEG at wk 2 and the gene signature of leukocytes was largely the same as wk 1 with a gene expression profile of B lymphocytes present in healthy cows and inflammatory cells present in metritis cows. We detected very few DEG for untreated healthy versus metritis cows at wk 4 (Table 1).

Based on the DEG, the circulating immune cells at wk 2 in antibiotic-treated metritis cows included monocytes, dendritic cells, and neutrophils, which were also found in untreated metritis cows at wk 2. However, antibiotic-treated metritis cows had DEG typically associated with NK cells and cytotoxic T cells. We propose that the presence of NK cells and cytotoxic T cells in antibiotic-treated cows represents an accelerated immunological timeline caused by the toxic effect of antibiotic on metritis pathogens (Jeon et al., 2021; Moraes et al., 2025).

Unexpectedly, there was a second spike in DEG (n = 1,541) indicative of a second wave of inflammation at wk 4 in the antibiotic-treated metritis cows (compared with antibiotic-treated healthy cows). The targeting and killing of infected or damaged cells by the NK cells and cytotoxic T cells at wk 2 may have released cytokines, pathogen-associated molecular patterns or damage-associated molecular patterns, or both, as inflammatory signals to recruit additional innate immune cells that were still in the circulation at 4 wk postpartum. Based on the low number of DEG (n = 12), we did not find evidence for this second wave of inflammatory cells in untreated cows (healthy versus metritis) cows at wk 4 (Table 1). We confirmed the nature of this second wave of inflammation at wk 4 by examining the overlap between gene lists over time (wk 1, wk 2, and wk 4) in healthy and metritis cows that were either untreated or antibiotic-treated (Supplemental Figure S1, see Notes). In untreated cows, the overlapping DEG for wk 1 to wk 2 indicated that the differences between healthy and metritis cows for B cell function and inflammation were present at wk 2 (Supplemental Figures S1A and S1B). There were few DEG for untreated healthy versus metritis cows at wk 4 (Table 1). In contrast, in antibiotic-treated cows, the overlapping DEG for wk 1 to wk 2 did not suggest B cell function nor inflammation (Supplemental Figures S1C and S1D). By wk 4, however, DEG associated with inflammation were present in the antibiotic-treated metritis cows and the gene list at wk 4 had a 17.8% overlap with the gene list from wk 1. We did not observe a similar overlap between wk 1 and wk 4 in antibiotic-treated healthy cows.

The observation that antibiotic treatment does not entirely recapitulate and even interferes with the “natural” progression of disease resolution may explain why clinical cure in an antibiotic-treated cow fails to improve pregnancy rates after treatment (Pereira et al., 2025). The accelerated immunological response at wk 2 postpartum and subsequent period of inflammation (wk 4 postpartum) observed in antibiotic-treated metritis cows may increase the clinical cure rate (reduce vaginal discharge score) by clearing the pathogens and restoring the integrity of the uterine tissue without ameliorating the true underlying causes of reduced fertility in metritis cows. We previously reported that the underlying cause of the reduced fertility later postpartum (when uterine inflammation has resolved) may be related to long-term changes in uterine function caused by metritis (Silva et al., 2024).

Two previous studies of metritis in postpartum cows have focused on the prepartum period through disease onset and resolution using flow cytometry (Bromfield et al., 2018; Casaro et al., 2023). Both studies reported greater numbers of B cells in healthy cows. The study of Casaro et al. (2023) reported that metritis cows had a greater number of monocytes that were less activated and a lesser number of neutrophils that were more activated compared with nonmetritis cows. We performed deconvolution by comparing the DEG of healthy versus metritis cows. Our approach is not quantitative with respect to the number of cells in circulation but more qualitative (i.e., we measured the function of immune cells but not the number of immune cells). Not directly measuring the number of immune cells using flow cytometry could be viewed as a limitation of this work. Measuring poly-A transcripts (mRNA-seq) instead of the entire transcriptome (total RNA seq) and the relatively small “n” within the comparison groups (5 to 6 cows) may have also reduced the sensitivity of our analyses. Nonetheless, using deconvolution of leukocyte RNA sequencing data, we show evidence for a cell-based inflammatory response in metritis cows, which agrees with previous publications (Sheldon et al., 2009; LeBlanc, 2020; Machado and Silva, 2020). The leukocyte RNA of healthy cows provided evidence for B cells, which are known to be abundant in postpartum cows (Cainelli et al., 2025).

In summary, resolution of postpartum metritis was accompanied by marked shifts in circulating immune cell function that differed substantially depending on antibiotic use. Whereas untreated metritis cows showed a progressive normalization of the blood leukocyte transcriptome by 4 wk postpartum, antibiotic-treated metritis cows followed a distinct trajectory characterized by cytotoxic T cell and NK cell transcriptomic signatures, followed by a secondary wave of inflammatory cell-associated gene expression. Such divergence in immune resolution may help explain the uncoupling between improved short-term clinical cure and inconsistent long-term reproductive outcomes.
